# Salinity Stress Is Beneficial to the Accumulation of Chlorogenic Acids in Honeysuckle (*Lonicera japonica* Thunb.)

**DOI:** 10.3389/fpls.2016.01563

**Published:** 2016-10-18

**Authors:** Kun Yan, Mingxing Cui, Shijie Zhao, Xiaobing Chen, Xiaoli Tang

**Affiliations:** ^1^Key Laboratory of Coastal Environmental Processes and Ecological Remediation, Yantai Institute of Coastal Zone Research, Chinese Academy of SciencesYantai, China; ^2^Northeast Institute of Geography and Agroecology, Chinese Academy of SciencesChangchun, China; ^3^State Key Laboratory of Crop Biology, Shandong Agricultural UniversityTai’an, China; ^4^School of Agriculture, Ludong UniversityYantai, China

**Keywords:** antioxidant capacity, medicinal plant, phenolics, photosynthesis, saline agriculture

## Abstract

Honeysuckle (*Lonicera japonica* Thunb.) is a traditional medicinal plant in China that is particularly rich in chlorogenic acids, which are phenolic compounds with various medicinal properties. This study aimed to examine the effects of salinity stress on accumulation of chlorogenic acids in honeysuckle, through hydroponic experiments and field trials, and to examine the mechanisms underlying the effects. NaCl stress stimulated the transcription of genes encoding key enzymes in the synthesis of chlorogenic acids in leaves; accordingly, the concentrations of chlorogenic acids in leaves were significantly increased under NaCl stress, as was antioxidant activity. Specifically, the total concentration of leaf chlorogenic acids was increased by 145.74 and 50.34% after 30 days of 150 and 300 mM NaCl stress, respectively. Similarly, the concentrations of chlorogenic acids were higher in the leaves of plants in saline, compared with non-saline, plots, with increases in total concentrations of chlorogenic acids of 56.05 and 105.29% in October 2014 and 2015, respectively. Despite leaf biomass reduction, absolute amounts of chlorogenic acids per plant and phenylalanine ammonia-lyase (PAL) activity were significantly increased by soil salinity, confirming that the accumulation of chlorogenic acids in leaves was a result of stimulation of their synthesis under salinity stress. Soil salinity also led to elevated chlorogenic acid concentrations in honeysuckle flower buds, with significant increases in total chlorogenic acids concentration of 22.42 and 25.14% in May 2014 and 2015, respectively. Consistent with biomass reduction, the absolute amounts of chlorogenic acid per plant declined in flower buds of plants exposed to elevated soil salinity, with no significant change in PAL activity. Thus, salinity-induced chlorogenic acid accumulation in flower buds depended on an amplification effect of growth reduction. In conclusion, salinity stress improved the medicinal quality of honeysuckle by promoting accumulation of chlorogenic acids, however, the mechanisms underlying this process were not consistent in flower buds and leaves. Honeysuckle appears to be a promising plant for cultivation in saline land. Our study deepens knowledge of medicinal plant ecology and may provide a guide for developing saline agriculture.

## Introduction

At present, it is a feasible way to develop saline agriculture with salt-tolerant economic plants for utilizing coastal saline land (Rozema et al., 2013; Panta et al., 2014; Qin et al., 2015). Salinity stress can interfere with metabolic processes in plant cells by inducing osmotic stress and ionic toxicity, leading to oxidative damage of biological macromolecules with generation of excess reactive oxygen species (Munns and Tester, 2008; Oukarroum et al., 2015). Phenolic compounds are secondary metabolites with high antioxidant capacity and play an important role in the protection of plants under abiotic stress against oxidative injury (Ahmad et al., 2010; Bose et al., 2014). Since phenolic compounds are primarily found in vacuoles, they are commonly considered to have secondary roles in antioxidant defense, in contrast to antioxidant enzymes and antioxidants in chloroplasts (Ferreres et al., 2011). To date, much attention has been paid to the investigation of phenolic compounds and their concentrations in plant tissues in the context of developing functional foods, cosmetics, and medicines, as some phenolic compounds have antimicrobial and anti-inflammatory qualities, in addition to their antioxidant activity (Tounekti and Munne-Bosch, 2012; Buhmann and Papenbrock, 2013; Routray and Orsat, 2014; Shahidi and Ambigaipalan, 2015; Sytar et al., 2016; Van Hung, 2016). Meanwhile, the ecological value of phenolic compounds in the adaption of plants to their environments has been relatively poorly explored.

There have been numerous reports that salt stress can induce the accumulation of phenolic compounds in plant tissues (Ksouri et al., 2007; Abrol et al., 2012; Lim et al., 2012; Colla et al., 2013; Shao et al., 2015; Zhao et al., 2015). However, the majority of these studies used hydroponic experimental methods, and there is little evidence of salinity-induced phenolic compound accumulation from field trials. Although hydroponic experiments facilitate the study of salt tolerance mechanisms in plants, they do not accurately reflect the ecological effects of plants in saline land (Tavakkoli et al., 2012). Notably, whether salinity-induced accumulation of phenolic compounds results from enhanced biosynthesis or is a phenomenon related to plant growth reduction remains ambiguous. Recently, a series of studies reported that the accumulation of secondary metabolites could be enhanced by deliberately applying drought stress, to improve the quality of products in medicinal plants (Selmar and Kleinwächter, 2013; Bloem et al., 2014; Kleinwaechter and Selmar, 2015). As with drought stress, salt stress also can increase reducing equivalents by depressing CO_2_ fixation, which likely facilitates the synthesis of highly reduced compounds, such as phenolic compounds. In fact, improved product quality has been evidenced in medicinal plants under hydroponic salt stress (Colla et al., 2013; Shao et al., 2015; Wang et al., 2016), and this is a potential advantage for the cultivation of medicinal plants in saline land. However, to the best of our knowledge, medicinal plants are unusually used for cultivation in saline land (Kushiev et al., 2005; Zhang et al., 2013).

Honeysuckle (*Lonicera japonica* Thunb.) is a traditional medicinal plant native to East Asia. Its flower bud and leaf are particularly rich in chlorogenic acids and have been used in Chinese medicine for many years. Chlorogenic acids, which are phenolic compounds, are the esters formed from quinic acid and caffeic acid. In honeysuckle, there are six kinds of caffeoylquinic acids: chlorogenic acid, neochlorogenic acid, cryptochlorogenic acid, isochlorogenic acid A, isochlorogenic acid B, and isochlorogenic acid C, which are named according to the position and number of the caffeic acid. In addition to antioxidant activity, chlorogenic acids also have anti-carcinogenic, anti-inflammatory, analgesic, antipyretic, anti-diabetic, antimicrobial, and antiviral functions (dos Santos et al., 2006; Wang et al., 2009; Gordon and Wishart, 2010; Xu et al., 2012), and have been widely used in the healthcare, food, and cosmetics industries (Zhang et al., 2008; Sytar et al., 2012; Sun et al., 2015). Increased concentrations of chlorogenic acids have been found in plants under hydroponic salt stress and in natural habitats with saline soil (Meot-Duros and Magne, 2009; Colla et al., 2013). In particular, Zhao et al. (2015) reported that hydroponic salt stress inhibited photosynthesis and increased leaf chlorogenic acid concentration in honeysuckle. However, only one kind of caffeoylquinic acid was considered in their study. Furthermore, it remains unknown whether saline land can improve the medicinal quality of honeysuckle by increasing concentrations of chlorogenic acids in flower buds and leaves.

Recently, we reported the selection of a salt tolerant honeysuckle cultivar and demonstrated its phytoremediation effect on saline soil (Yan et al., 2015, 2016). In this paper, we focus on the medicinal quality of honeysuckle, and dissect the responses and mechanisms of accumulation of chlorogenic acids under salinity stress, using both hydroponic experiments and field trials. We hypothesized that salinity stress could improve the medicinal quality of honeysuckle by promoting the synthesis of chlorogenic acids. Our study can provide new insight into the interaction between plants and saline soil and contribute to the understanding of saline agriculture in coastal zones.

## Materials and Methods

### Hydroponic Experimental Conditions

Bare-rooted honeysuckle plants were planted in plastic pots filled with quartz sand in April 2015. The plants were watered with Hoagland solution (pH 5.7) and placed in a climatic chamber (Qiushi, China). The photon flux density was approximately 400 μmol m^-2^ s^-1^ (12 h per day from 07:00 to 19:00), and day/night temperature and humidity in the chamber were controlled at 25°C/18°C and 65%, respectively. After 60 days, healthy and uniform plants were selected for salt treatment. NaCl was added to nutrient solution incrementally by daily 50 mM steps, to final concentrations of 150 and 300 mM. Nutrient solution without added NaCl was used for the cultivation of control plants. After salt stress for 15 and 30 days, the newly expanded leaves from the middle of a shoot were sampled from four replicate plants and separated into two portions. One portion was used for measurement of relative expression levels of genes encoding hydroxycinnamoyl-CoA quinate: hydroxycinnamoyl transferase, and PAL family proteins. The other portion was dried at 40°C to a constant weight and ground to pass through a 0.25-mm sieve for measurement of the concentrations of chlorogenic acids.

### Field Trial Conditions

The experiment site was established in Dongying Halophyte Arboretum, Dongying Academy of Agricultural Sciences, Shandong province, China (37°24′N, 118°39′E and 8.8 m above sea level). This area has a warm temperate continental monsoon climate. The annual average temperature and precipitation at this site are 12.8°C and 555.9 mm, respectively.

Bare-rooted honeysuckle plants were planted in a non-saline area of the arboretum in November 2013. Four replicate plots (3 m × 4 m) were constructed in non-saline and saline areas. The initial soil nutrients, salinity, and pH were reported in our recent study (Yan et al., 2016). The average electronic conductance and sodium adsorption ratios were 486 μs cm^-1^ and 9.51, respectively, in non-saline plots and 910 μs cm^-1^ and 16.43, respectively, in saline plots. To avoid border effects, an isolation belt of 0.5 m was left around the plots. The plots were plowed, and compound fertilization was applied at 750 kg ha^-1^. In April 2014, 45 plants were transplanted to each plot; plant and row spacing were 0.5 m and 0.75 m, respectively. Flower buds were collected from three randomly selected plants in each plot in late May 2014 and 2015, and leaves were collected in October of the same years. Flower buds and leaves were dried at 40°C to a constant weight and ground to pass through a 0.25-mm sieve to measure concentrations of chlorogenic acids, DPPH scavenging rate, and stable isotopic composition. Another sample of flower buds and canopy expanded leaves was used to measure MDA content and PAL activity. The total concentration of chlorogenic acids was defined as the sum of the concentrations of chlorogenic acid, neochlorogenic acid, cryptochlorogenic acid, and isochlorogenic acids A, B, and C. The absolute amount of chlorogenic acids per plant was calculated as the total concentration of chlorogenic acids × dry mass.

### Measurement of the Concentration of Chlorogenic Acids

**FIGURE 1:**
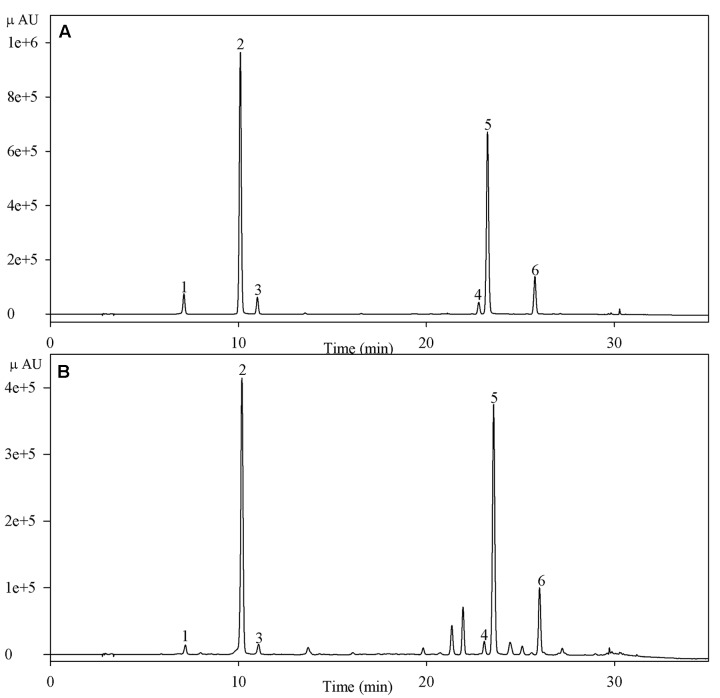
**High performance liquid chromatogram of chlorogenic acids of standards **(A)** and a sample **(B)** detected at 330 nm.** Peaks 1–6 indicate neochlorogenic acid, chlorogenic acid, cryptochlorogenic acid, and isochlorogenic acids B, A, and C, respectively.

Dry plant powder (0.1 g) was homogenized in 8 ml 60% (v/v) methanol, and ultrasonic extraction was carried out at 40°C for 40 min. The mixture was centrifuged for 10 min at 10000 × *g*. Chlorogenic acid concentrations were assayed according to the method of Li et al. (2014) with some modification. The assay was performed in a high performance liquid chromatography system (Thermo, USA) with a Hypersil C18 column (4.6 mm × 150 mm; particle size, 5.0 μm). The supernatant was filtered through a 0.45 μm membrane filter before injecting into the column; the injection volume was 10 μl. The mobile phase consisted of 0.3% (v/v) formic acid aqueous solution (A) and acetonitrile (B), and a gradient elution program was applied as follows: at 0 min, the volume ratio between A and B was 95/5, and the ratio was changed to 77/23 from 0 to 26 min, subsequently to 10/90 from 26 to 32 min, and finally returned to 95/5 at 35 min through a linear gradient. The flow rate was 1.0 ml min^-1^, and the column temperature was 38°C. Chlorogenic acids in samples were identified by comparing their retention times in UV spectra with those of the standards (**Figure 1**). Chlorogenic acids were detected at 330 nm, and their concentrations were determined using a standard curve plotted using known concentrations of chlorogenic acids.

### Determination of Antioxidant Activity

Plant extract diluted in methanol (0.3 ml) and pure methanol (0.3 ml) were each mixed with 60 μM DPPH methanol solution (3 ml). The mixture was vigorously shaken and incubated for 30 min in the dark at room temperature, and then the absorbance was measured at 517 nm (Khan et al., 2013). The DPPH scavenging rate (%) was calculated as (A_blank_ – A_sample_)/A_blank_ × 100, where A_sample_ and A_blank_ were the absorbance of mixtures in the presence and absence of plant extract, respectively.

### Measurement of MDA Content

Plant tissues (0.5 g) were ground under liquid nitrogen and then homogenized in 5 ml of 50 mM potassium phosphate buffer (pH 7.8). After centrifugation at 4°C, 13000 × *g*, for 10 min, the supernatant was prepared for measurement of MDA content, according to the methods of Yan et al. (2010).

### Measurement of PAL Activity

Plant tissues (0.2 g) were ground under liquid nitrogen and then homogenized in 5 ml of 50 mM borate buffer (pH 8.8) containing 20 mM β-mercaptoethanol, 5% (v/w) PVP, 1 mM EDTA-Na_2_ and 0.1% (v/w) Triton X-100. The homogenate was centrifuged at 10000 × *g*, 4°C for 20 min, and the supernatant was collected for enzyme assay. To determine PAL activity, 0.2 ml of supernatant was mixed with 2.8 ml 0.02 M L-phenylalanine (dissolved in 50 mM borate buffer at pH 8.8), and incubated for 1 h at 30°C. The increase in OD_290 nm_ due to the formation of cinnamic acid was measured according to the protocol described by Khan et al. (2013). One unit of PAL activity was defined as an increase of 0.1 in the OD_290 nm_ h^-1^ mg^-1^ protein. Protein concentration was estimated by staining with coomassie brilliant blue, with bovine serum albumin as a standard.

### Real-Time Quantitative PCR Analysis

For real time gene expression analysis, PCR reactions contained 1 μl of diluted cDNA (10 ng), 10 μl of SYBR Green PCR Master Mix (Applied Biosystems, USA) and 200 nM of specific primers in a final volume of 20 μl. Actin (*ACT1*) (GenBank Accession No. GQ241342) was used as an internal reference gene to calculate relative transcript levels. The primers for *ACT1. HQT. PAL1. PAL2*, and *PAL3* are listed in **Table 1**. All PCRs were performed using a Fast Real Time PCR System (Applied Biosystems, USA) under the following conditions: 2 min at 95°C, and 40 cycles of 15 s at 95°C and 60 s at 60°C in optical 96-well reaction plates. The specificity of amplicons was verified by melting curve analysis. Three technical replicates were analyzed for each gene.

**Table 1:** Primers for real time quantitative PCR.

Gene	Sense primer	Antisense primer
*ACT1*	CCAGTATTGTAGGTAGACCAAGAC	TCAATGGGGTATTTCAAGGTAAGG
*HQT*	CGAGCAAGTTATACATAGC	AGTTGTGGATTCTCTTAGC
*PAL1*	GCCAATCCAGTCACTAACC	CGTAAATTCTCCTCCAAATGC
*PAL2*	GCTCGCCCTTGTTAATGG	GTGGTGCTTCAACTTATGC
*PAL3*	TGAACGCTGGAATCTTTGG	GGTGATGTTGTGGTTGAGG


**HQT*, hydroxycinnamoyl-CoA quinate: hydroxycinnamoyl transferase; *PAL*, phenylalanine ammonia-lyase (the PAL gene family consists of *PAL1. PAL2*, and *PAL3*). *ACT1* was used as an internal reference gene.*

### Stable Carbon and Oxygen Isotopic Composition

For ^13^C/^12^C analysis, samples (0.06 mg) were packed in tin capsules and then combusted in an Elemental Analyzer (Flash EA^TM^ 1112, Thermo Scientific, Bremen, Germany). The CO_2_ evolving from flash combustion of the samples (in the presence of O_2_ at a temperature of 900°C) was flushed into an isotope ratio mass spectrometer (Finnigan Delta Plus XP^TM^, Thermo Scientific, Bremen, Germany). For ^18^O/^16^O analysis, samples (0.06 mg) were packed in silver capsules and dropped into a pyrolyser (Finnigan TC/EA^TM^, Thermo Scientific, Bremen, Germany), where the CO_2_ and H_2_ produced at a temperature of 1450°C without oxygen were sent to a gas chromatography column and then to the mass spectrometer. Isotopic composition was denoted using the standard delta notation (δ, ‰):δ(‰) = *R*_sample_/*R*_stand_ – 1, where *R*_sample_ and *R*_stand_ were isotopic ratios in samples and standards, respectively. Carbon and oxygen standards were Vienna Pee Dee Belemnite and Vienna Mean Ocean Water.

### Statistical Analyses

One-way ANOVAs were carried out using SPSS 16.0 (SPSS Inc., Chicago, IL, USA) for all data sets. The values presented are the means of samples collected from four replicate plants in hydroponic experiments and four replicate plots in field trials. Comparisons of means were performed using a least significant difference test, and differences were considered significant at *P* < 0.05. Regression analysis between the DPPH scavenging rate and total concentration of leaf chlorogenic acids was carried out using SPSS 16.0 to determine their correlation.

## Results

### Concentration of Chlorogenic Acids and Antioxidant Activity in Leaves of Honeysuckle Plants Under NaCl Stress

**FIGURE 2:**
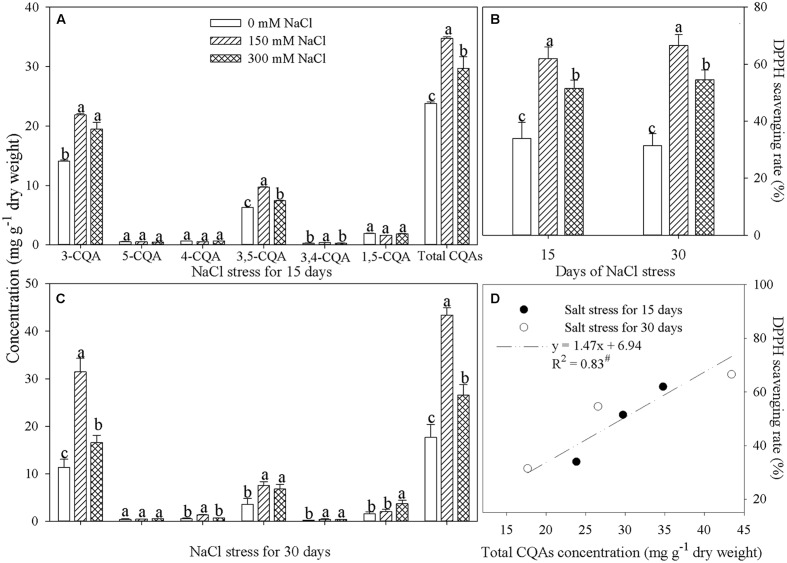
**Concentration of chlorogenic acids **(A,C)**, DPPH scavenging rate **(B)**, and regression analysis **(D)** in the leaves of honeysuckle under hydroponic NaCl stress.** Chlorogenic acid, neochlorogenic acid, cryptochlorogenic acid, isochlorogenic acids A, B, and C, and total chlorogenic acids are indicated by 3-CQA, 5-CQA, 4-CQA, 3,5-CQA, 3,4-CQA, 1,5-CQA, and total CQAs, respectively. Data are expressed as the means of four replicate experiments (±SD). Different letters on error bars indicate salt-induced significant differences (*P* < 0.05). The significant correlation (*P* < 0.05) is indicated by #.

Leaf chlorogenic acid and isochlorogenic acid A concentrations were much higher than those of other chlorogenic acids, and increased significantly after 15 and 30 days of NaCl stress (**Figures 2A,C**). As a result of the increases in these compounds, total concentration of chlorogenic acids in leaves was remarkably elevated by NaCl stress (**Figures 2A,C**). DPPH scavenging rate can be used to indicate antioxidant activity (Lim et al., 2012). Under NaCl stress, the DPPH scavenging rate increased significantly in honeysuckle leaves (**Figure 2B**), and a significant positive correlation was identified between the DPPH scavenging rate and the total concentration of chlorogenic acids (**Figure 2D**).

### Gene Transcription in Leaves of Honeysuckle Plants Under NaCl Stress

**FIGURE 3:**
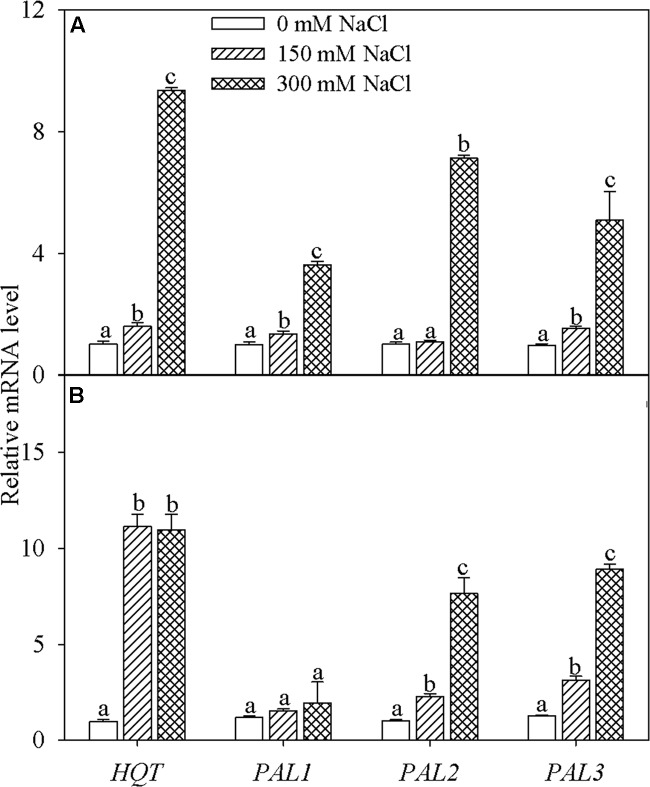
**Hydroxycinnamoyl-CoA quinate: hydroxycinnamoyl transferase (*HQT*) and phenylalanine ammonia-lyase (PAL) family gene transcription in the leaves of honeysuckle plants under hydroponic NaCl stress for 15 **(A)** and 30 **(B)** days.** The PAL gene family consists of *PAL1. PAL2*, and *PAL3*. Data are expressed as the means of four replicate experiments (±SD). Different letters on error bars indicate salt-induced significant differences (*P* < 0.05).

Transcription of *HQT* was markedly enhanced in honeysuckle leaves subjected to 15 and 30 days of NaCl stress (**Figure 3**). PAL gene family consists of *PAL1. PAL2*, and *PAL3*. After 15 days of 150 mM NaCl stress, transcription of *PAL1* and *PAL3* was significantly elevated, while expression of all three genes (*PAL1. PAL2*, and *PAL3*) was significantly elevated after exposure to 300 mM NaCl salt stress for 15 days (**Figure 3A**). After 30 days, transcription of *PAL2* and *PAL3* was significantly increased in leaves of plants exposed to both 150 and 300 mM NaCl (**Figure 3B**).

### Accumulation of Chlorogenic Acids, Biomass, Antioxidant Activity, PAL Activity, MDA Content, and Isotopic Composition of Leaves of Honeysuckle Plants Exposed to Soil Salinity

Concentrations of chlorogenic acid, cryptochlorogenic acid, and isochlorogenic acids A, B, and C in leaves were significantly higher in plants cultivated in saline plots compared with those in non-saline plots, and a remarkable increase in the concentration of total chlorogenic acids in leaves was observed, with increases of 56.05 and 105.29% in October 2014 and 2015, respectively (**Table 2**). Despite the significant decrease in leaf dry weight per plant due to soil salinity, the absolute amount of total chlorogenic acids per plant was also remarkably elevated, with increases of 11.49 and 25.83% in plants in saline plots in October 2014 and 2015, respectively (**Table 2**). Soil salinity led to a significant increase in the DPPH scavenging rate and PAL activity, while there was no obvious effect on the MDA content of leaves (**Table 2**). Stable carbon (δ^13^C) and oxygen (δ^18^O) isotopic composition were significantly higher in leaves of plants cultivated in saline plots, compared with those in non-saline plots in October 2014 and 2015 (**Table 2**).

**Table 2:** Concentrations of chlorogenic acids, biomass, antioxidant capacity, phenylalanine ammonia-lyase (PAL) activity, malondialdehyde (MDA) content, carbon (δ^13^C) and oxygen (δ^18^O) isotopic composition, and absolute amount of total chlorogenic acids per plant (ATC) in the leaves of honeysuckle plants in non-saline and saline plots in October 2014 and 2015.

	October 2014		October 2015	
		
	Non-saline	Saline	Non-saline	Saline
3-CQA (mg g^-1^ DW)	6.19 ± 1.55b	12.29 ± 2.34a	8.68 ± 1.31b	16.58 ± 3.14a
5-CQA (mg g^-1^ DW)	0.62 ± 0.15a	0.77 ± 0.17a	0.10 ± 0.01a	0.11 ± 0.01a
4-CQA (mg g^-1^ DW)	0.50 ± 0.18b	0.80 ± 0.16a	0.29 ± 0.01b	0.48 ± 0.08a
3,5-CQA (mg g^-1^ DW)	5.87 ± 0.64b	8.67 ± 0.44a	8.13 ± 1.04b	17.49 ± 2.36a
3,4-CQA (mg g^-1^ DW)	0.43 ± 0.23b	0.47 ± 0.07a	0.44 ± 0.06b	0.70 ± 0.12a
1,5-CQA (mg g^-1^ DW)	2.38 ± 0.49b	3.82 ± 0.35a	1.45 ± 0.36b	3.86 ± 0.72a
Total CQAs (mg g^-1^ DW)	16.95 ± 2.20b	26.45 ± 2.53a	19.09 ± 3.61b	39.19 ± 7.33a
DPPH scavenging rate (%)	44.80 ± 4.33b	69.79 ± 6.34a	49.80 ± 5.81b	75.54 ± 5.64a
PAL activity (mg g^-1^ protein)	12.02 ± 3.20b	20.40 ± 2.20a	17.25 ± 3.53b	28.63 ± 3.68a
MDA content (nmol g^-1^ FW)	54.97 ± 7.01a	50.93 ± 8.01a	40.19 ± 4.01a	44.98 ± 5.21a
Dry weight (g plant^-1^)	51.98 ± 4.12a	36.94 ± 2.99b	78.16 ± 8.02a	50.23 ± 6.55b
ATC (g plant^-1^)	0.87 ± 0.05b	0.97 ± 0.03a	1.51 ± 0.17b	1.9 ± 0.20a
δ^13^C (‰)	-27.76 ± 0.19b	-26.72 ± 0.25a	-28.50 ± 0.23b	-26.79 ± 0.63a
δ^18^O (‰)	23.60 ± 0.18b	24.81 ± 0.39a	24.59 ± 0.26b	25.33 ± 0.28a


*Data are expressed as the mean of four replicate plots (±SD). Within each row, different letters indicate significant differences (*P* < 0.05) at the same sampling time. DW and FW indicate dry and fresh weight, respectively. Chlorogenic acid, neochlorogenic acid, cryptochlorogenic acid, isochlorogenic acids A, B, and C, and total chlorogenic acids are indicated by 3-CQA, 5-CQA, 4-CQA, 3,5-CQA, 3,4-CQA, 1,5-CQA, and total CQAs, respectively.*

### Accumulation of Chlorogenic Acids, Biomass, Antioxidant Activity, PAL Activity, and MDA Content in Honeysuckle Flower Buds Exposed to Soil Salinity

In May 2014, the concentrations of chlorogenic acids were significantly increased in flower buds of plants exposed to soil salinity, with significant increases in concentrations of chlorogenic acid, cryptochlorogenic acid, and isochlorogenic acids A and B in May 2015 (**Table 3**). There was a significant increase in the total concentration of chlorogenic acids in flower buds of 22.42 and 25.14% in May 2014 and 2015, respectively. Cultivation in saline soil also led to an increase in the DPPH scavenging rate in flower buds in May 2014, and a significant increase in this parameter in May 2015 (**Table 3**). No significant changes in MDA content or PAL activity were observed in flower buds of plants exposed to soil salinity (**Table 3**). Consistent with the decrease in dry weight per plant of those grown in saline plots, the absolute amount of total chlorogenic acids per plant in flower buds was also decreased in May 2014 and 2015 (**Table 3**).

**Table 3:** Concentrations of chlorogenic acids, biomass, antioxidant capacity, phenylalanine ammonia-lyase (PAL) activity, malondialdehyde (MDA) content, and absolute amount of total chlorogenic acids per plant (ATC) in flower buds of honeysuckle plants in non-saline and saline plots in May 2014 and 2015.

	May 2014		May 2015	
		
	Non-saline	Saline	Non-saline	Saline
3-CQA (mg g^-1^ DW)	23.69 ± 1.15b	27.69 ± 1.65a	23.72 ± 1.61b	28.25 ± 1.95a
5-CQA (mg g^-1^ DW)	0.70 ± 0.04a	0.67 ± 0.05a	0.69 ± 0.07a	0.74 ± 0.09a
4-CQA (mg g^-1^ DW)	0.46 ± 0.03b	0.57 ± 0.04a	0.45 ± 0.04b	0.56 ± 0.02a
3,5-CQA (mg g^-1^ DW)	10.19 ± 2.23a	14.57 ± 2.03a	15.01 ± 2.13b	20.55 ± 2.61a
3,4-CQA (mg g^-1^ DW)	0.33 ± 0.02a	0.36 ± 0.05a	0.33 ± 0.02b	0.41 ± 0.04a
1,5-CQA (mg g^-1^ DW)	2.31 ± 0.17a	2.14 ± 0.21a	2.22 ± 0.19a	2.60 ± 0.23a
Total CQAs (mg g^-1^ DW)	37.69 ± 3.02b	46.14 ± 3.29a	42.44 ± 4.22b	53.11 ± 4.82a
DPPH scavenging rate (%)	50.46 ± 3.22a	58.31 ± 4.12a	52.96 ± 3.01b	61.83 ± 3.86a
PAL activity (U g^-1^ protein)	43.14 ± 4.37a	40.14 ± 2.37a	41.14 ± 3.37a	45.35 ± 5.37a
MDA content (nmol g^-1^ FW)	25.85 ± 2.37a	24.01 ± 1.73a	30.56 ± 2.479a	28.78 ± 2.41a
Dry weight (g plant^-1^)	1.91 ± 0.36a	1.21 ± 0.23b	3.81 ± 0.46a	2.36 ± 0.37b
ATC (mg plant^-1^)	71.37 ± 9.19a	53.81 ± 6.21a	161.09 ± 18.78a	124.87 ± 20.58a


*Data are expressed as the mean of four replicate plots (±SD). Within each row, different letters indicate significant differences (*P* < 0.05) at the same sampling time. DW and FW indicate dry and fresh weight, respectively. Chlorogenic acid, neochlorogenic acid, cryptochlorogenic acid, isochlorogenic acids A, B, and C, and total chlorogenic acids are indicated by 3-CQA, 5-CQA, 4-CQA, 3,5-CQA, 3,4-CQA, 1,5-CQA, and total CQAs, respectively.*

## Discussion

Honeysuckle is widely distributed throughout the world due to its great environmental adaptability (Evans, 1984; Schierenbeck, 2004). In agreement with the results of Zhao et al. (2015), a significant increase in leaf chlorogenic acid concentration was observed in honeysuckle plants under NaCl stress (**Figures 2A,C**). Furthermore, in addition to its effects on chlorogenic acid, NaCl stress also remarkably elevated concentrations of isochlorogenic acids A and B (**Figures 2A,C**). The antioxidant activity of chlorogenic acid may protect plants against oxidative damage under environmental stress (Sytar et al., 2012), and increased chlorogenic acid concentration is often noted, along with enhanced antioxidant ability, in plants exposed to salinity stress, suggesting a potential correlation between these variables (Meot-Duros and Magne, 2009; Colla et al., 2013). Consistent with this hypothesis, leaf antioxidant activity in honeysuckle plants was also induced by NaCl stress, as indicated by the increase in the DPPH scavenging rate. Moreover, the significant positive correlation between the concentration of chlorogenic acids and the DPPH scavenging rate confirms that accumulation of leaf chlorogenic acids assisted in strengthening antioxidant protection in plants exposed to NaCl stress (**Figures 2B,D**).

The concentrations of chemical compounds indicate the amount based on leaf mass, and its value reflects the extent of accumulation in plant tissue. To date, studies have attached importance to the accumulation of defense chemicals in plants under salt stress, however, they have generally neglected the synthesis of these chemicals (Ksouri et al., 2007; Falleh et al., 2012; Lim et al., 2012; Petridis et al., 2012; Baatour et al., 2013; Colla et al., 2013; Zhao et al., 2015; Wang et al., 2016), possibly leading to an incomplete understanding of their physiological responses. HQT and PAL are key enzymes in the pathway of chlorogenic acids synthesis (Niggeweg et al., 2004). NaCl stress stimulated the synthesis of leaf chlorogenic acids by increasing transcription of *HQT* and PAL family genes in honeysuckle (**Figure 3**). The enhanced synthesis contributed to the accumulation of chlorogenic acids and might represent increased generation of reduction power as a consequence of salt-induced inhibition of carbon assimilation (Yan et al., 2015).

Through a 2 years field trial in coastal saline land, we demonstrated that soil salinity was beneficial to the accumulation of chlorogenic acids in leaves, as indicated by the significant increase in their concentrations (**Table 2**). Plant growth is commonly decreased by salinity stress, due to the high sensitivity of photosynthesis to this environmental variable (Kalaji et al., 2011; Yan et al., 2013). Stable carbon and oxygen isotopic composition can be used to indicate the long term water use efficiency and stomatal conductance of plants (Moreno-Gutierrez et al., 2012). Higher levels of δ^18^O and δ^13^C in leaves of honeysuckle plants in saline plots suggests that soil salinity enhanced water use efficiency by reducing leaf stomatal conductance and consequently, stomatal limitation on photosynthesis could lead to an inhibition on biomass accumulation (**Table 2**). However, the increased concentration of leaf chlorogenic acids did not stem entirely from the amplification effect of biomass reduction, because soil salinity also caused a significant elevation of the absolute amount of chlorogenic acids per plant in leaves (**Table 2**). In addition, the significant increase in PAL activity in leaves indicates improved synthesis of phenolic compounds in response to soil salinity (**Table 2**). Thus, similar to NaCl stress, soil salinity can also enhance the synthesis of chlorogenic acids in leaves.

The energy and carbon assimilated by photosynthesis is primarily consumed for cell maintenance, with the remainder allocated to plant growth and the synthesis of defensive chemicals (Munns and Gilliham, 2015). Under optimal growth conditions, plants may grow at a higher rate, with lower accumulation of defense chemical compounds (Hofmann and Jahufer, 2011). Similarly, there appears to be a trade-off between plant growth and the synthesis of phenolic compounds in honeysuckle plants grown on saline land. However, according to Neilson et al. (2013), enhanced synthesis of chlorogenic acids can be considered as a cost-effective strategy, as it may alleviate growth reduction by protecting against salinity-induced oxidative damage. Rozema and Schat (2013) also argued that there was no trade-off between plant growth reduction and high salt tolerance, because marsh halophytes had a stronger salt tolerance and a similar growth rate, compared with inland glycophytes. Consistent with the accumulation of chlorogenic acids, leaf antioxidant activity was enhanced to protect against oxidative injury, as indicated by the significant increase in the DPPH scavenging rate and lack of a significant difference in MDA content in plants in saline plots compared with those in non-saline plots (**Table 2**). Therefore, the accumulation of leaf chlorogenic acids may be an important ecological mechanism in the acclimation of honeysuckle to saline land.

Soil salinity also reduced biomass and increased concentrations of chlorogenic acids in honeysuckle flower buds (**Table 3**). Flower buds are carbon sinks and require a supply of carbohydrate for growth and metabolism. Thus, the reduction in biomass of flower buds might be caused by the decline in carbon supply, due to the inhibition of leaf photosynthesis under salinity stress. Soil salinity did not activate the biosynthesis of chlorogenic acids in flower buds, as the absolute amount of chlorogenic acids was decreased in flower buds of plants grown in saline plots, compared with those in non-saline plots (**Table 3**). In addition, the lack of obvious change in PAL activity also suggests that the biosynthesis of phenolic compounds in flower buds was not induced by soil salinity (**Table 3**). Therefore, the increase in the concentration of chlorogenic acids in flower buds was a consequence of salinity-induced growth reduction. Correlated with the elevated concentration of chlorogenic acids, antioxidant activity was also enhanced to presumably protect against lipid peroxidation in flower buds of plants grown under saline stress, as indicated by the increase in the DPPH scavenging rate and lack of significant change in MDA content (**Table 3**). However, the enhanced antioxidant activity in flower buds was not a positive protection response to salinity stress, because the increase in the concentrations of chlorogenic acids was passive. Notably, in consistence with the finding of more phenolic concentration in flower buds than leaves in honeysuckle (Sandigawad, 2015), a greater concentration of chlorogenic acids was observed in flower buds than leaves in this study (**Tables 2** and **3**), implying the superior medicinal value of flower buds.

## Conclusion

Irrespective of the absolute amount of chlorogenic acids, salinity stress enhanced the medicinal quality of honeysuckle by increasing the concentration of these compounds in flower buds and leaves. In agreement with our hypothesis, stimulation of biosynthesis contributed to the accumulation of chlorogenic acids in leaves under salinity stress. In contrast, the increased concentrations of chlorogenic acids in flower buds were a consequence of the amplification effect of growth reduction. In combination with its phytoremediation effect on saline soil (Yan et al., 2016), honeysuckle is a promising species for cultivation in saline land. The next task will be to improve the production of honeysuckle in saline land by strengthening its salt tolerance. Genetic engineering is a feasible way to improve plant salt tolerance, and the selection of promising genes should be a priority (Rozema and Schat, 2013). Our findings indicate that both biomass accumulation and product quality may be improved by overexpression of key genes encoding enzymes in the chlorogenic acid synthesis pathway, including *HQT* and PAL family genes.

## Author Contributions

KY designed the experiments, performed data analysis, and wrote the manuscript. MC participated in experimental design and polished the language. SZ reviewed the manuscript and made some critical suggestions. XC provided the necessary plant material and assisted in construction of the experimental plots. XT participated in conducting the experiments and performed data analysis.

## Conflict of Interest Statement

The authors declare that the research was conducted in the absence of any commercial or financial relationships that could be construed as a potential conflict of interest.

## Abbreviations

DPPH: diphenylpicrylhydrazyl radical
HQT: hydroxycinnamoyl-CoA quinate: hydroxycinnamoyl transferase
MDA: malondialdehyde
PAL: phenylalanine ammonia-lyasexs
